# Supplementary material for: Rpn11-mediated ubiquitin processing in an ancestral archaeal ubiquitination system
Source: Nat Commun. 2018 Jul 12;9:2696. doi: 10.1038/s41467-018-05198-1 (PMC6043591; doi:10.1038/s41467-018-05198-1)
Supplement: Supplementary file 1 — Supplementary Information [file 41467_2018_5198_MOESM1_ESM.pdf]

Rpn11-mediated ubiquitin processing in an ancestral archaeal ubiquitination system

Fuchs et al.

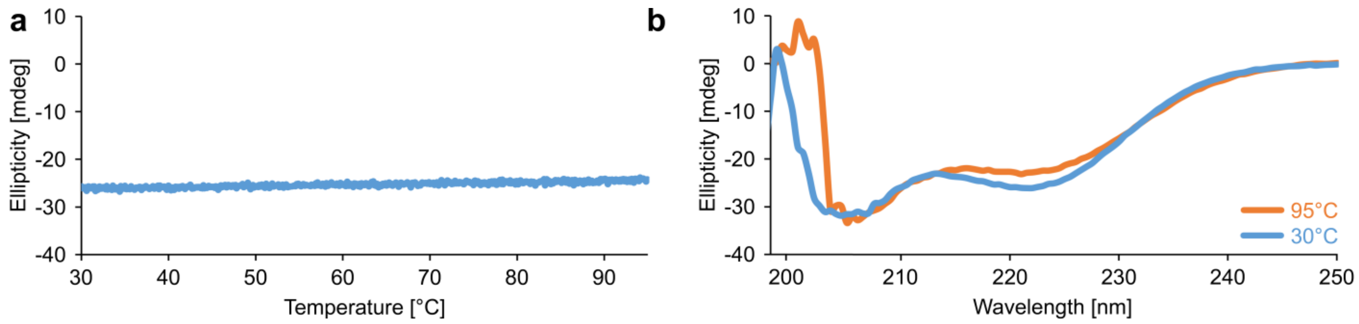

Supplementary Figure 1. CsUb is a thermostable protein.

Shown are CD melting curve (**a**) and CD spectra of CsUb at 30°C and 95°C (**b**).

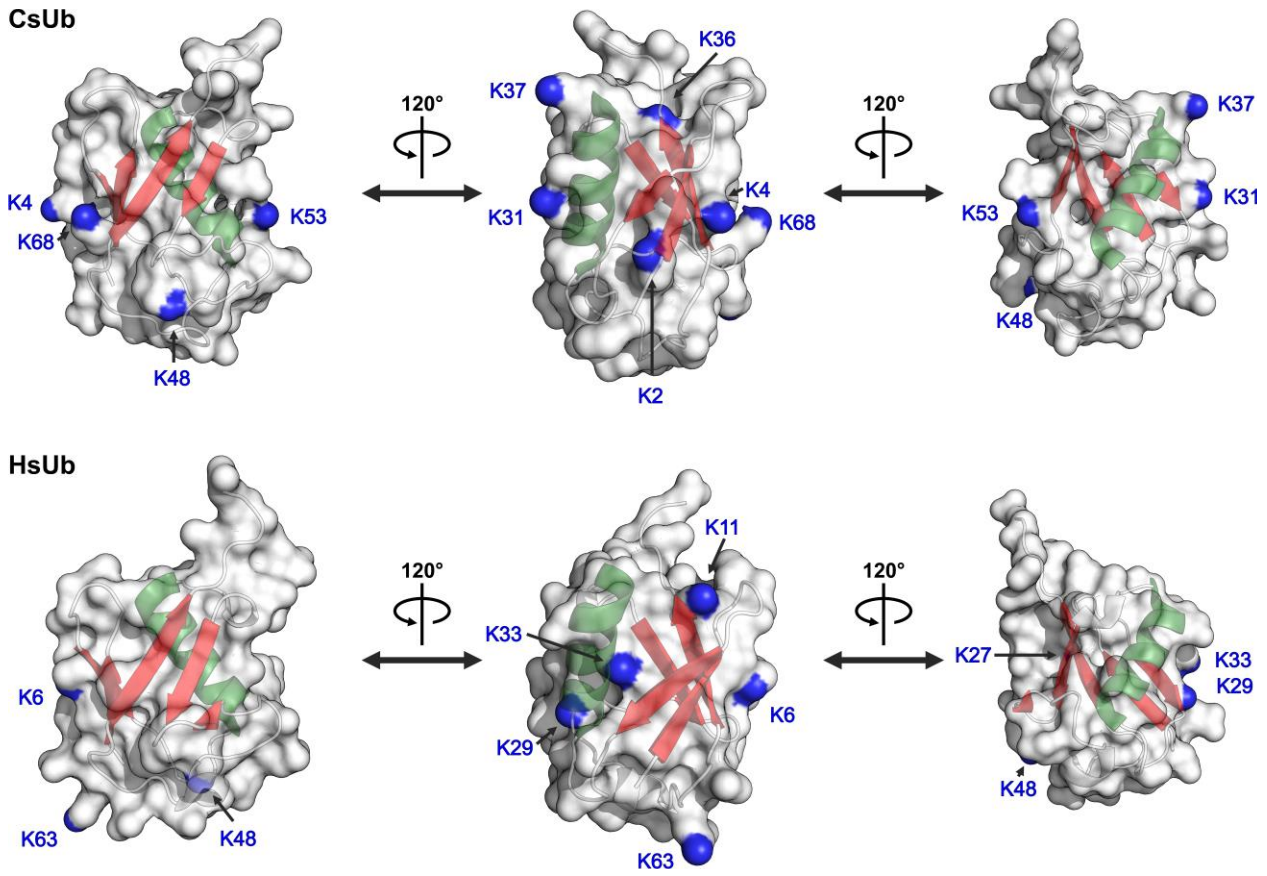

Supplementary Figure 2. Position of lysine residues in CsUb and human Ub (HsUb).

Except for Lys31, all CsUb lysines are found at different surface locations compared to HsUb.

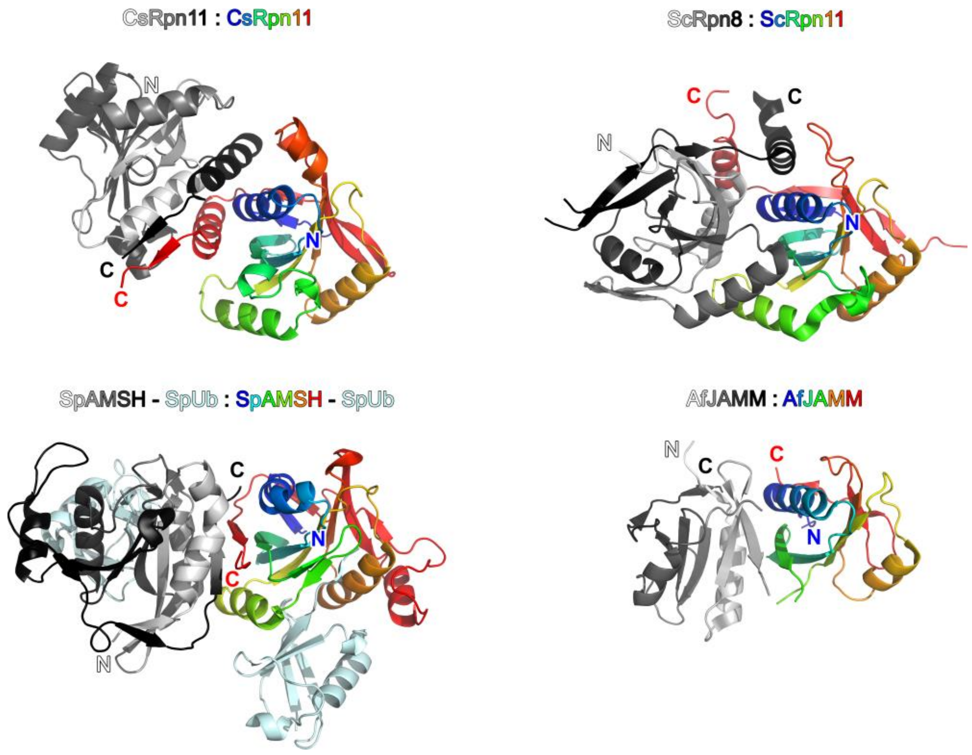

Supplementary Figure 3. Comparison of crystallographic JAMM dimers.

Shown are dimeric structures of CsRpn11 (PDB 6FJU), *S. pombe* AMSH - Ub (SpAMSH - SpUb; PDB 4K1R [1]), *S. cerevisiae* Rpn8$^{\Delta180\text{-}306}$ - Rpn11$^{\Delta240\text{-}306}$ (ScRpn8:Rpn11; PDB 4O8X [2]) and *A. fulgidus* JAMM1 (AfJAMM; PDB 1R5X [3]). The rainbow-colored subunit is shown in the same orientation for each structure, highlighting the divergent orientations of the second subunit in grayscale. While ScRpn8-ScRpn11 dimerizes as seen in EM-reconstructions of the proteasome (PDB 5T0C [4]), the other JAMM proteins exist as monomers in solution (see Supplementary Figure 2) [1, 3].

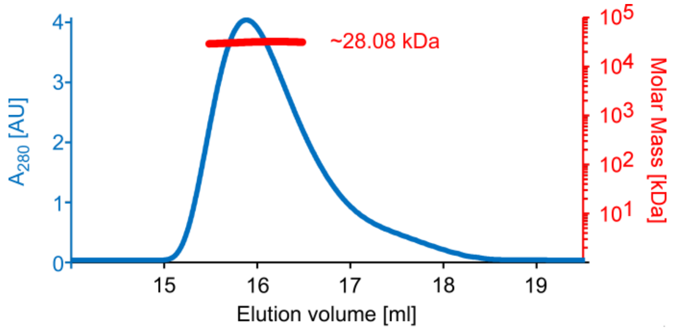

Supplementary Figure 4. Oligomerization state of CsRpn11 determined by static light scattering.

CsRpn11 was subjected to size-exclusion chromatography combined with static light scattering (SEC-MALS). Data (red) were determined for the UV peak area (blue). The determined mass, 28.08 kDa, is in the range of the expected size for a single CsRpn11 protein chain (24.5 kDa), consistent with a monomeric state in solution.

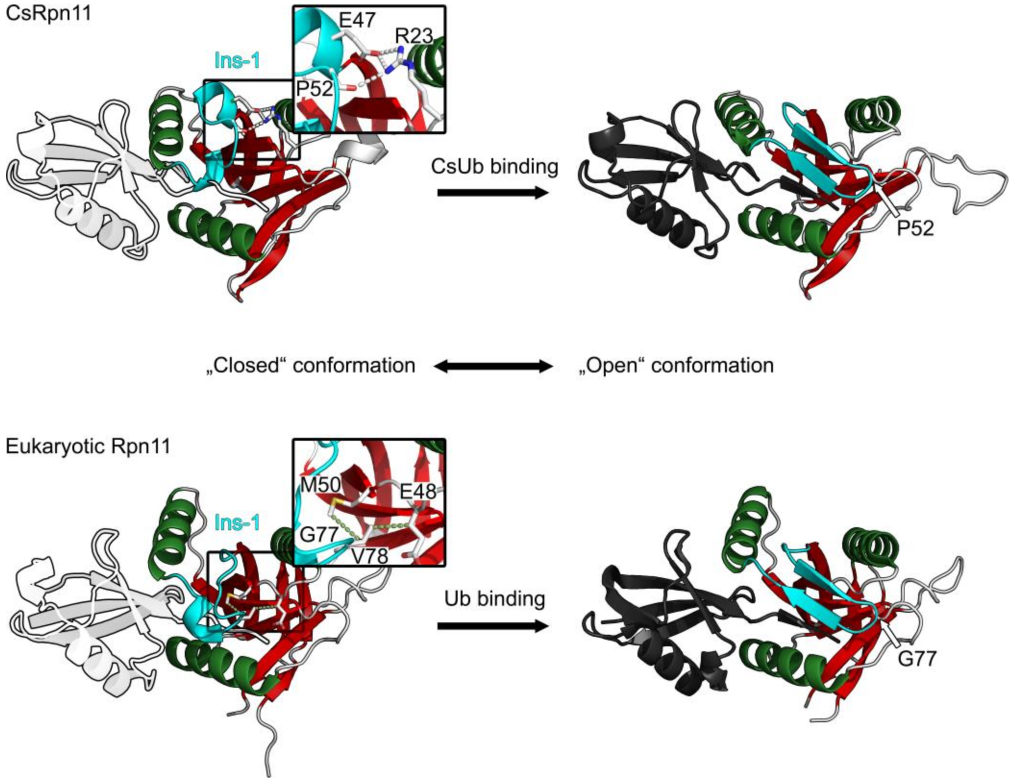

Supplementary Figure 5. CsUb binding induces an evolutionary conserved conformational switch in CsRpn11 Ins-1.

Shown are unliganded (left) and Ub-bound (right) structures of CsRpn11 (PDB 6FJU and 6FNN) and eukaryotic Rpn11 (PDB 4O8X and 5U4P), colored as in Fig. 3. In Ub free structures, the Ins-1 element (cyan) adopts a “closed” conformation that is sterically incompatible with Ub binding (shown in greyscale). Contacts that stabilize this conformation are highlighted in insets [5].

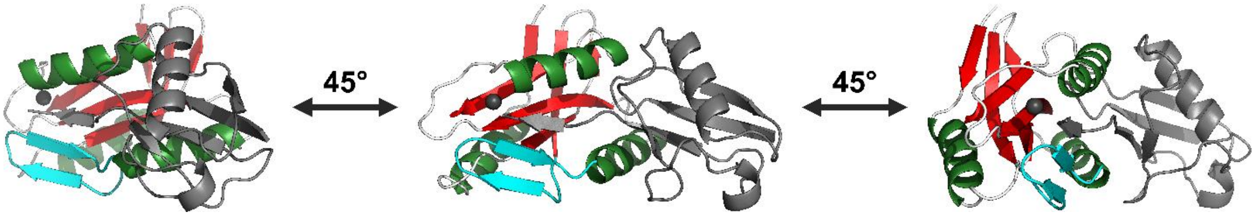

Supplementary Figure 6. The CsRpn11$^{\Delta 149\text{-}202}$-CsUb co-structure shown from different angles.

The C-terminus of bound CsUb (grey) comes to lie precisely at the CsRpn11 (rainbow colored) cleavage site.

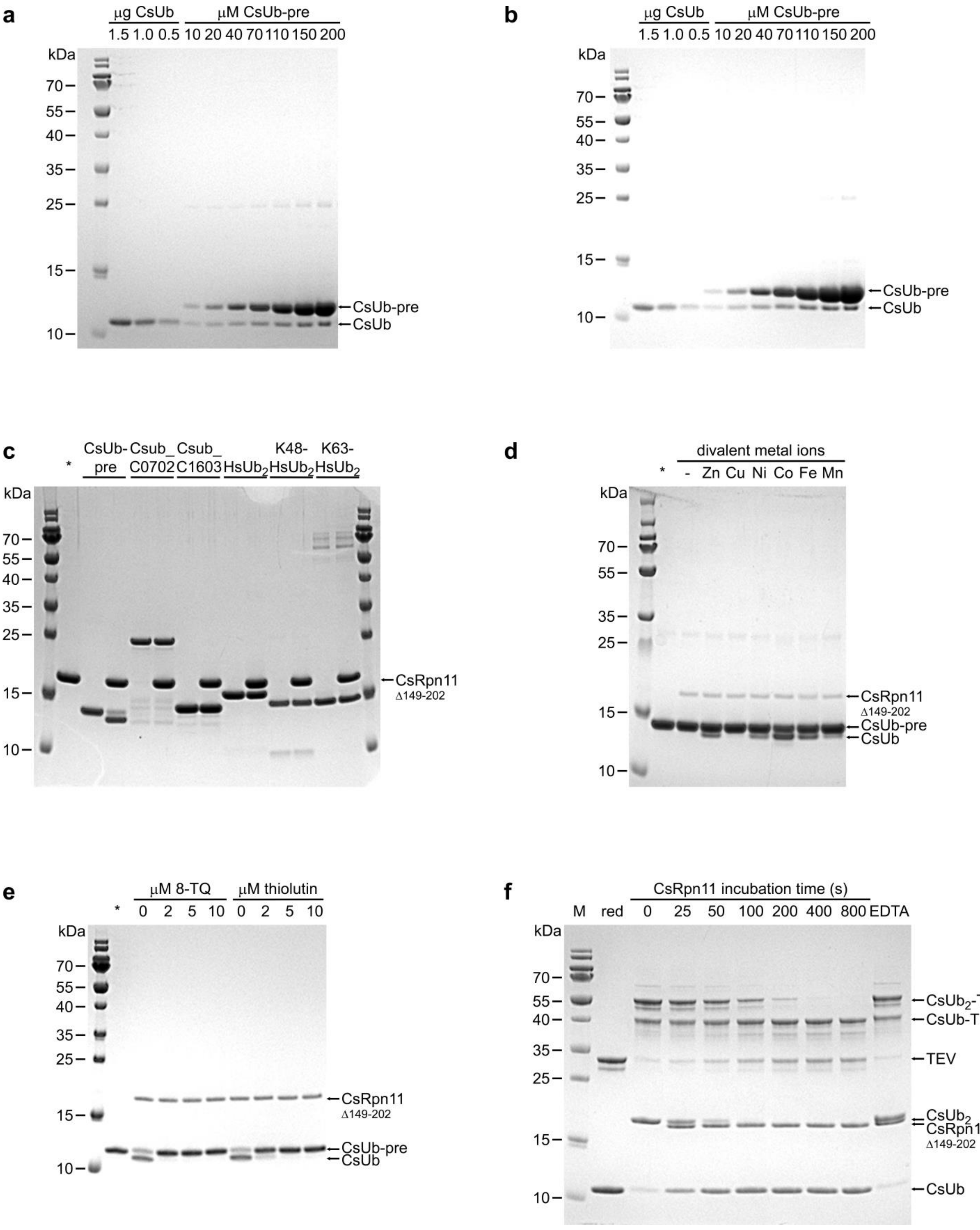
a
μg CsUb
μM CsUb-pre
1.5 1.0 0.5 10 20 40 70 110 150 200
kDa
70
55
40
35
25
15
10
CsUb-pre
CsUb
b
μg CsUb
μM CsUb-pre
1.5 1.0 0.5 10 20 40 70 110 150 200
kDa
70
55
40
35
25
15
10
CsUb-pre
CsUb
c
*
CsUb-pre
Csub_C0702
Csub_C1603
HsUb2
K48-HsUb2
K63-HsUb2
kDa
70
55
40
35
25
15
10
CsRpn11 Δ149-202
d
divalent metal ions
* - Zn Cu Ni Co Fe Mn
kDa
70
55
35
25
15
10
CsRpn11 Δ149-202
CsUb-pre
CsUb
e
μM 8-TQ
μM thiolutin
* 0 2 5 10 0 2 5 10
kDa
70
55
40
35
25
15
10
CsRpn11 Δ149-202
CsUb-pre
CsUb
f
CsRpn11 incubation time (s)
M red 0 25 50 100 200 400 800 EDTA
kDa
70
55
40
35
25
15
10
CsUb2-TEV
CsUb-TEV
TEV
CsUb2
CsRpn11 Δ149-202
CsUb

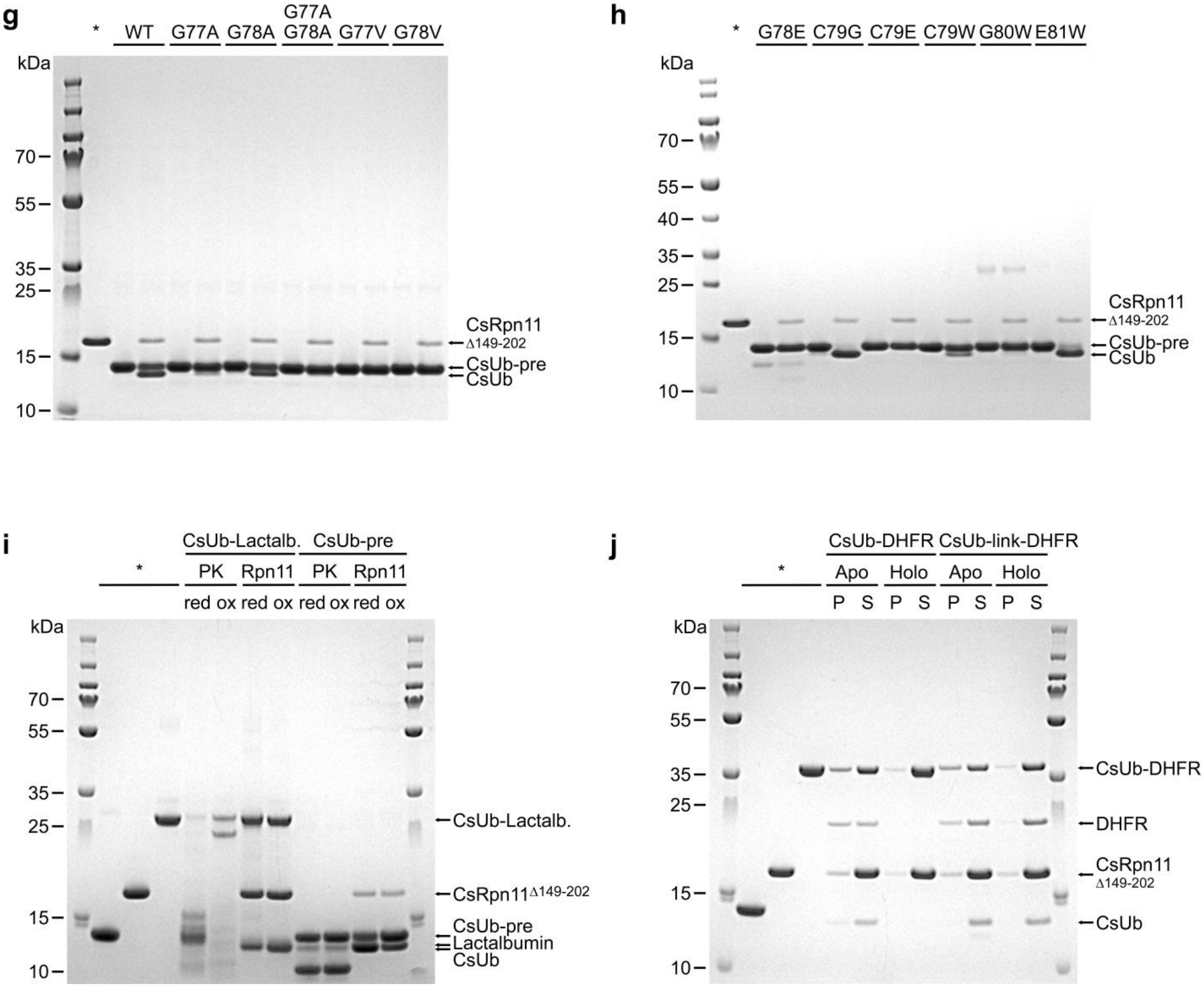

Supplementary Figure 7. Full gels used for generation of main paper figures.

Shown are the complete gels used in Fig. 4c (**a**, **b**), 4e (**c**), 5a (**d**), 5c (**e**), 6 (**f**), 7a (**g**, **h**), 7b (**i**) and 7c (**j**). Asterisks mark CsRpn11$^{\Delta 149\text{-}202}$ (**c**, **i**, **j**), CsUb-pre (**d**, **e**, **i**, **j**), CsUb-Lactalbumin (**i**) or CsUb-DHFR (**j**), which are shown for comparison.

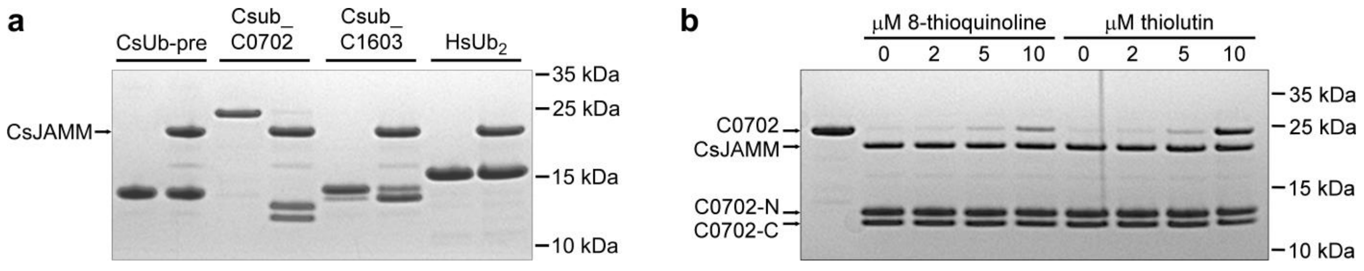

Supplementary Figure 8. CsJAMM substrate specificity and response to inhibitors 8-thioquinoline and thiolutin.

a SDS-PAGE analysis of CsJAMM substrate processing. CsJAMM cleaves *C. subterraneum* SAMP precursors Csub_C0702 and Csub_C1603, but is inactive towards CsUb-pre and linearly linked human diubiquitin ($HsUb_2$). Each substrate is shown before (left) and after (right) treatment with CsJAMM. **b** Inhibitor effects on CsJAMM activity. Processing of SAMP Csub_C0702 was tested in the presence of increasing concentrations of 8-TQ [6] and thiolutin [7] resulting in N- (C0702-N) and C-terminal (C0702-C) SAMP fragments.

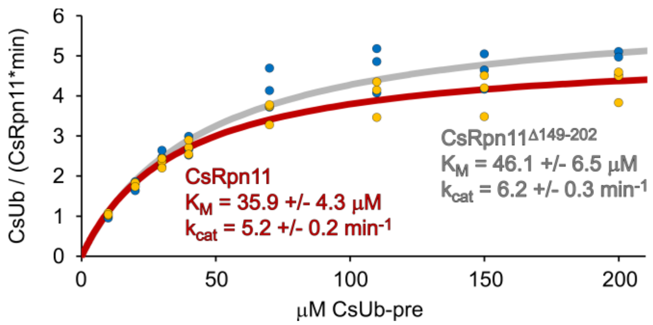

Supplementary Figure 9. Kinetics of CsUb-pre processing by full-length CsRpn11 and CsRpn11$^{\Delta 149\text{-}202}$ at 60°C.

The Michaelis-Menten plot is based on three independent experiments.

Supplementary Table 1. Structural comparison of Ub-like proteins.

	CsUb	HsUb	HvSAMP1	HvSAMP2
**HsUb**	12.2			
**HvSAMP1**	6.9	6.7		
**HvSAMP2**	5.4	5.7	4.2	
**PfSAMP2:PfJAMM1**	5.4	5.0	5.6	4.8

The structural similarities of CsUb (PDB 6FJ7), *H. sapiens* Ub (HsUb, PDB 4XOF), *H. volcanii* SAMP1 (HvSAMP1, PDB 3PO0), *H. volcanii* SAMP2 (HvSAMP2, PDB 4HRS), and *P. furiosus* SAMP2 in context of the PfSAMP2:PfJAMM1 complex (PDB 5LDA_B) are expressed by DALI Z-scores [8]. Greater structural similarity results in higher Z-Scores.

Supplementary Table 2. Structural comparison of the JAMM domain in selected proteins.

	CsRpn11	ScRpn11	HsCSN5	BRCC36	HsAMSH
**ScRpn11**	21.2				
**HsCSN5**	19.2	19.8			
**CfBRCC36**	19.7	18.5	16.4		
**HsAMSH**	15.4	14.8	12.4	16.0	
**PfJAMM1**	15.8	16.6	14.1	17.3	16.5

The structural similarities of the CsRpn11 (PDB 6FJU_A), *S. cerevisiae* Rpn11 (ScRpn11, PDB 4OWP_B), *H. sapiens* CSN5 (HsCSN5, PDB 4F7O_A), *C. floridanus* BRCC36 (CfBRCC36, PDB 5CW3_A), *H. sapiens* AMSH (HsAMSH, PDB 3RZU_A), and *P. furiosus* JAMM1 (PDB 5LD9_B) JAMM domains, expressed by DALI Z-scores [8].

Supplementary Table 3. Codon-optimized gene sequences used in this study

Gene	Sequence
Csub_C1473 **(CsRpn11)**	ATGCGAGTGCGTATATACCCGCTTGCGTTGGCTAAGGTCGTCAAGCACGCCGCGTCTAGTCTGC AGCGTGAAGTTGCAGGGTTGCTGGTCGGCAAGAGCGCTGGAAAAGTCCTCGAAATTTGGGATG CGGTCACCGGCGAACAGTATGGGACTCCTGCGTATGTGCAGCTTGATGAGATGGTTATGGCTAA GGTGGCCGAGGAATTGTCGAAATCCGATAAAAACTTATACATTGTTGGTTGGTACCATTCCCAT CCCGGACTCGACGTATTTCTGAGCCCGACCGACATTGACACACAAAAACGCTATCAAGCCATGT TCTCTAAAGCGGTAGCACTAGTGGTTGATCCGGTAGATTATGCAAAAACCCGGCGCATCAGTA GTTTAAAATTCAAAGTGTTTCAGATCTCAAAAGAAGGCCGTGTTGTGAGCCTGCCAGTGTCTAT TGGCGTGCATCGCGCGAAATTACTGGAATCCACGTTCCACGCCCTGAGCACGTTTGATTTTATG CACATCCTGGGCGAATCAAGCGGTAAAACCCGCGACAAACCGCTGTCGGAAGAGCAGGAGTCG CTGCTGGGTAAAGCAAAAAAACTGTTTGGTGCCTAA
Csub_C1474 **(CsUb-pre)**	ATGAAAATTAAAATTGTACCCGCCGTGGGTGGAGGGAGCCCTCTCGAACTTGAAGTAGCTCCA AACGCGACCGTGGGTGCGGTGCGTACCAAGGTCTGTGCGATGAAAAAGTTGCCGCCGGATACA ACGCGCTTAACTTATAAAGGGCGCGCTCTGAAAGATACCGAGACTCTGGAGTCGCTGGGCGTC GCAGATGGTGACAAATTTGTTCTGATCACCCGTACGGTTGGCGGCTGCGGCGAACCGATCCGCC GGGCCGCATAA
Csub_C0703 **(CsJAMM)**	ATGAAACTGGTTGTTAAACGTCTGGTGTTTGAGGAAATTGCCAAACGCTGCATCGAAGGCTACC CGTACGAAACCGCCGGTCTGATGTTCGGCGATCTGGGTAACCGCCTGGTGCTGGACATCTACCC GGTGCAAAACATTCACGAACAGGACCGTCGCGTGCGTTACCGTATTGATCCGATGGAATACTAT CGCGCGGAGAAAACCGCGGAGGAGAAAGGCATGACGATTGTTGGTGTTTATCACAGCCATCCG AATGTTGCGGCCCGCCCGAGCGCCTATGATCTGGAGTATGCGCTGCCGCCGTGGAGCTATCTGA TCCTGAGCATCAACCATGAAAAAGTGCTGGAATATACGAGCTGGCGTGCGGTGCAGAATAATG GCGAAAAAAAATTTATTCAGGAAGAAGTGGTGATCGAATAA
Csub_C0702	ATGGCTGTGAAGGTTTATCTACCGACTCCTTTACGGCAGTACGCCGATGGTCGCGATATGGTAG AGTTAGATGGTTCCACAGTAGGTGAAGTTCTGAATAAATTGGTTTCTCGTTATACCGCCCTGCA GAAGCATCTGTTTAATGAAAATGGGGCGATTCGCTCGTTCGTTAACGTCTTTGTAAATAACGAG GATATCCGCTTTCTCGAAGGCGTCAACACCAAAATTAAAGATGGAGATGTGGTGTACATTATCC CGAGTATTGCGGGGGGTCTTTCAATCGCAGCGCCCGCTGCTGTGGCAAAAAAGCTGGGCCGTA CCGTCAAACAACACGGCCGTATCACTGTCCCAGCTAAACTGCTCAAAAAAGCGAAAAAAAACG AAGTGACCGTGATCATAGACGACGTGAAATATATCTTTGAACCGGATCGTTATAACCGCATTTA CCTGCCACCGACGCTGCGCGAAAAAATTGCGCATCTTAGCTCGTTCGAATTCACGCTGAGCGAC GGCGAACTGATTCTCAGATTTCGTCGCTTCTAA
Csub_C1603	ATGGCCGGCGGCCGTTTAAAAATTCTGACTAAGTACTATGCGGTATTGCGTGAACGCGTCGGCA AAGCTAGTGAAGAATTCGAACTACCGCAAGGCTCGACCGTAATTGACTTTCTGGAAAAGCTGC GTCAGGTTTACGGCGGCGTGCTGGGGGATTTGTTCGAGGGGGATGGACTTCGAACCGGTTTTGC CCTTGCACTCAACGGAGAGAGCCTCGATCGGAAATTATGGGCGTCTACACGCCTGAAAGACGG TGATGTGGTTGTGGTCCTGCCGCCTATCGCGGGTGGTTATCTGAAACTGGGTTCCCTGACGCCA CGCTGGCCGTAA

Supplementary Table 4. Oligonucleotides used in this study

Primer	Sequence
**CsRpn11-fwd**	CGATACCATATGGCATCAGCTACAGCACGAGTGCGTATATACCCGCTTG
**CsRpn11$^{\Delta 145\text{-}202}$-rvs**	GAGCATCTCGAGAAGCTTAGCTCACAACACGGCCTTC
**CsRpn11$^{\Delta 149\text{-}202}$-rvs**	GAGCATCTCGAGAAGCTTAAGACACTGGCAGGCTCAC
**CsRpn11$^{\Delta 167\text{-}202}$-rvs**	GAGCATCTCGAGAAGCTTACGTGCTCAGGGCGTGG
**CsRpn11$^{\Delta 175\text{-}202}$-rvs**	GAGCATCTCGAGAAGCTTATTCGCCCAGGATGTGCATAAAATC
**CsUb-fwd**	CGATACCATATGAAAATTAAAATTGTACCCGCCGTG
**CsUb-pre^{G77A}-rvs**	GCTATGCTCGAGAAGCTTATGCGGCCCGGCGGATCGGTTCGCCGCAGCCGGCAACCGTACGGGTGATCAGAAC
**CsUb-pre^{G78A}-rvs**	GCTATGCTCGAGAAGCTTATGCGGCCCGGCGGATCGGTTCGCCGCATGCGCCAACCGTACGGGTG
**CsUb-pre$^{G77A/G78A}$-rvs**	GCTATGCTCGAGAAGCTTATGCGGCCCGGCGGATCGGTTCGCCGCATGCTGCAACCGTACGGGTGATCAGAAC
**CsUb-pre^{G77V}-rvs**	GCTATGCTCGAGAAGCTTATGCGGCCCGGCGGATCGGTTCGCCGCAGCCCACAACCGTACGGGTGATCAG
**CsUb-pre^{G78V}-rvs**	GCTATGCTCGAGAAGCTTATGCGGCCCGGCGGATCGGTTCGCCGCACACGCCAACCGTACGGGTG
**CsUb-pre^{G78E}-rvs**	GCTATGCTCGAGAAGCTTATGCGGCCCGGCGGATCGGTTCGCCGCATTCGCCAACCGTACGGGTG
**CsUb-pre^{C79G}-rvs**	GCTATGCTCGAGAAGCTTATGCGGCCCGGCGGATCGGTTCGCCGCCGCCGCCAACCGTACGG
**CsUb-pre^{C79E}-rvs**	GCTATGCTCGAGAAGCTTATGCGGCCCGGCGGATCGGTTCGCCCTCGCCGCCAACCGTACGG
**CsUb-pre^{C79W}-rvs**	GCTATGCTCGAGAAGCTTATGCGGCCCGGCGGATCGGTTCGCCCCAGCCGCCAACCGTAC
**CsUb-pre^{G80W}-rvs**	GCTATGCTCGAGAAGCTTATGCGGCCCGGCGGATCGGTTCCCAGCAGCCGCCAACCGTAC
**CsUb-pre^{E81W}-rvs**	GCTATGCTCGAGAAGCTTATGCGGCCCGGCGGATCGGCCAGCCGCAGCCGCCAACC
**CsUb^{C33A}-fwd**	GCCGCGATGAAAAAGTTGCCGCCG
**CsUb^{C33A}-rvs**	GACCTTGGTACGCACCGCAC
**CsUb-rvs**	GCCGCCAACCGTACGG
**DHFR-fwd**	CCGTACGGTTGGCGGCTTGAACTGCATCGTCGCC

**linker-DHFR-fwd**	CCGTACGGTTGGCGGCTGCGGTGAAATGGTTCGACCATTGAACTGC
**DHFR-rvs**	GCTATGCTCGAGAAGCTTAGTCTTTCTTCTCGTAGACTTCAAAC
**Lactalbumin Fragment 1-fwd**	CCGTACGGTTGGCGGCACAAAATGTGAGGTGTTCCGGG
**Lactalbumin Fragment 2-fwd**	CTCTGCCGGAATGGGTTTGCACCACCTTCCACACCTCTGGTTACGACACCCAGGCTATCG TTCAGAACAACGACTCTACCGAATACGGTCTGTTCCAGATCAACAACAAAATCTGGTGC
**Lactalbumin Fragment 3-fwd**	CCAGATCAACAACAAAATCTGGTGCAAAGACGACCAGAACCCGCACTCTTCTAACATCT GCAACATCTCTTGCGACAAATTCCTGGACGACGACCTGACCGACGACATCATGTGCG
**Lactalbumin Fragment 4-fwd**	CCGACGACATCATGTGCGTTAAAAAAATCCTGGACAAAGTTGGTATCAACTACTGGCTG GCTCACAAAGCTCTGTGCTCTGAAAAACTGGACCAGTGGCTGTGCGAAAAACTGTAAGC
**Lactalbumin-rvs**	GCTATGCTCGAGAAGCTTACAGTTTTTCGCACAGCC
**GyrA-fwd**	CCGTACGGTTGGCGGCTGCATCACGGGAGATGCACTAG
**GyrA-rvs**	GCTATGAAGCTTAAACCACGCCGTTTTGGTC

Supplementary Table 5. Crystallization conditions and cryo protection

Protein	Protein buffer	Reservoir solution (RS)	Cryo-protectant
**CsUb**	25.6 mg ml^{-1} CsUb,<br>20 mM Tris-HCl pH 8.0,<br>250 mM NaCl	100 mM sodium acetate pH 4.5,<br>200 mM lithium sulfate,<br>50% (v/v) PEG 400	-
**CsRpn11**	12 mg ml^{-1} CsRpn11,<br>20 mM Tris-HCl pH 8.0,<br>50 mM NaCl,<br>1 M urea	100 mM HEPES-NaOH pH 7.0,<br>15% (w/v) PEG 4000	RS + 20% glycerol
**CsRpn11 $^{\Delta 149-202}$**	8.5 mg ml^{-1} CsRpn11 $^{\Delta 149-202}$,<br>20 mM HEPES-NaOH pH 7.5,<br>250 mM NaCl,<br>1 M urea, 2 mM EDTA	100 mM sodium acetate pH 4.6,<br>1.5 M ammonium sulfate,<br>25% (w/v) PEG 4000	RS + 30% glycerol
**CsRpn11$^{\Delta 149-202}$-CsUb**	6 mg ml^{-1} CsRpn11 $^{\Delta 149-202}$,<br>3.3 mg ml^{-1} CsUb,<br>20 mM HEPES-NaOH pH 7.5, 150 mM NaCl	100 mM sodium acetate pH 4.6,<br>1.5 M ammonium sulfate	RS + 15% (v/v) PEG200

Supplementary Table 6. Data collection and refinement statistics (molecular replacement)

	**CsUb**	**CsRpn11**	**CsRpn11$^{\Delta 149\text{-}202}$**	**CsRpn11$^{\Delta 149\text{-}202}$-CsUb**	**CsRpn11$^{\Delta 149\text{-}202}$-CsUb, zinc soak**
**Data collection**					
Space group	$P2_12_12_1$	$P2_12_12_1$	$P4_12_12$	$C222_1$	$C222_1$
Cell dimensions					
a, *b*, *c* (Å)	29.5, 42.8, 51.4	64.5, 65.6, 99.7	39.0, 39.0, 194.0	84.7, 94.6, 170.9	84.2, 94.8, 171.0
α, β, γ (°)	90, 90, 90	90, 90, 90	90, 90, 90	90, 90, 90	90, 90, 90
Resolution (Å)	32.9 – 1.05 (3.13 – 1.05)	33.8 – 1.65 (1.75 – 1.65)	38.2 – 1.35 (1.43 – 1.35)	37.9 – 1.85 (1.96 – 1.85)	37.8 – 1.95 (2.06 – 1.95)
R_{merge}	0.051 (0.627)	0.055 (0.973)	0.066 (1.11)	0.074 (0.779)	0.081 (1.03)
$I / \sigma I$	22.3 (2.18)	18.8 (1.77)	21.1 (2.25)	19.8 (2.66)	14.4 (1.70)
Completeness (%)	98.2 (89.0)	99.7 (98.2)	100 (99.9)	99.6 (97.9)	99.7 (98.3)
Redundancy	11.0 (5.10)	6.86 (6.29)	12.4 (11.5)	12.9 (12.9)	6.90 (6.88)
**Refinement**					
Resolution (Å)	32.9 – 1.05 (1.08 – 1.05)	33.8 – 1.65 (1.69 – 1.65)	38.2 – 1.35 (1.38 – 1.35)	37.9 – 1.85 (1.89 – 1.85)	37.8 – 1.95 (2.00 – 1.95)
No. reflections	29058	49099	32583	55970	47811
R_{work} / R_{free}	15.3 / 19.5 (32.0 / 33.0)	16.6 / 19.0 (29.5 / 31.1)	17.1 / 21.0 (36.7 / 41.8)	17.3 / 20.1 (39.8 / 39.2)	17.7 / 20.1 (39.8 / 40.3)
No. atoms	752	3044	1345	4088	3939
Protein	666	2792	1196	3708	3603
Zn^{2+}	-	2	-	2	2
SO_4	10	5	10	10	25
Water	76	245	139	368	309
B-factors	16.3	34.5	20.2	42.3	45.3
Protein	14.9	33.9	18.2	41.7	44.6
Ligand/ion	22.7	53.9	61.1	53.1	58.8
Water	28.6	41.0	34.3	48.6	51.6
R.m.s. deviations					
Bond lengths (Å)	0.020	0.020	0.015	0.010	0.010
Bond angles (°)	2.34	1.89	1.73	1.39	1.31
**PDB code**	**6FJ7**	**6FJU**	**6FJV**	**6FNN**	**6FNO**

Values in parentheses are for highest-resolution shell.

Supplementary References

1. Shrestha RK*, et al.* Insights into the mechanism of deubiquitination by JAMM deubiquitinases from cocrystal structures of the enzyme with the substrate and product. *Biochemistry* **53**, 3199-3217 (2014).

2. Worden EJ, Padovani C, Martin A. Structure of the Rpn11-Rpn8 dimer reveals mechanisms of substrate deubiquitination during proteasomal degradation. *Nat Struct Mol Biol* **21**, 220-227 (2014).

3. Ambroggio XI, Rees DC, Deshaies RJ. JAMM: a metalloprotease-like zinc site in the proteasome and signalosome. *PLoS Biol* **2**, E2 (2004).

4. Chen S*, et al.* Structural basis for dynamic regulation of the human 26S proteasome. *Proc Natl Acad Sci U S A* **113**, 12991-12996 (2016).

5. Worden EJ, Dong KC, Martin A. An AAA Motor-Driven Mechanical Switch in Rpn11 Controls Deubiquitination at the 26S Proteasome. *Mol Cell* **67**, 799-811 e798 (2017).

6. Perez C*, et al.* Discovery of an Inhibitor of the Proteasome Subunit Rpn11. *J Med Chem* **60**, 1343-1361 (2017).

7. Lauinger L*, et al.* Thiolutin is a zinc chelator that inhibits the Rpn11 and other JAMM metalloproteases. *Nat Chem Biol* **13**, 709-714 (2017).

8. Holm L, Kaariainen S, Rosenstrom P, Schenkel A. Searching protein structure databases with DaliLite v.3. *Bioinformatics* **24**, 2780-2781 (2008).
